# Assessment of Psychotic Risk in a Sample of Children and Adolescents with Autism Spectrum Disorder Compared to a Group of “Clinical High Risk” Patients: A Preliminary Study

**DOI:** 10.3390/children11030372

**Published:** 2024-03-21

**Authors:** Valeria Mammarella, Elena Monducci, Alessia Maffucci, Letizia Terenzi, Mauro Ferrara, Carla Sogos

**Affiliations:** 1 Department of Psychology, Sapienza University of Rome, 00185 Rome, Italy; valeria.mammarella@uniroma1.it; 2 Department of Human Neuroscience, Sapienza University of Rome, 00185 Rome, Italy; elena.monducci@uniroma1.it (E.M.); alessia.maffucci@uniroma1.it (A.M.); letizia.terenzi@uniroma1.it (L.T.); mauro.ferrara@uniroma1.it (M.F.)

**Keywords:** autism, children, adolescent, psychosis, risk, evaluation, clinical high risk

## Abstract

1. Background: Autism spectrum disorder and psychotic risk show several overlapping symptoms, so differential diagnosis is often difficult. In addition, there is a high rate of comorbidity between the two conditions, which further complicates the work of clinicians. We evaluated the presence of subthreshold psychotic symptoms and/or defined psychotic risk syndromes in autistic children and adolescents; we compared the prevalence, type, and severity of psychotic risk symptoms with those of a group of non-autistic patients at clinical high risk for psychosis (CHR-P). 2. Methods: In total, 23 autistic patients and 14 CHR-P patients without autism (aged 8–17) were enrolled in the study. The main assessment was made through clinical interviews for autism (Autism Diagnostic Observation Schedule, Second Edition—ADOS-2, Autism Diagnostic Interview, Revised—ADI-R) and psychotic risk (Schizophrenia Proneness Instrument, Child and Youth version—SPI-CY, Structured Interview for Psychosis Risk Syndromes—SIPS). 3. Results: No above-threshold psychotic risk symptoms were detected in our autistic patients, but subthreshold psychotic symptoms were identified in all areas. Specific items from all four dimensions of SIPS appear to be more specific for psychotic risk than autism without comorbidity. 4. Conclusions: An a priori screening of psychotic risk in neurodiverse populations is fundamental to prevent more severe conditions. Research should clarify the effective specificity of the available tools to modify them to improve their detection capability.

## 1. Introduction

Since the first nosographic classifications of mental disorders, there has always been a broad debate on the association between autism spectrum disorders and schizophrenia spectrum disorders, currently conceptualized as two distinct conditions in the latest nosographic manuals [1].

Autism spectrum (AS) disorder is a neurodevelopmental condition characterized by repetitive patterns of behavior and interests and problems in social interactions, whose symptoms are present from early childhood and affect the daily functioning of individuals [2]. Schizophrenia spectrum disorders, instead, are chronic remitting and disruptive disorders associated with significant abnormalities and the progressive deterioration of a wide variety of cognitive, psychosocial, vocational, and behavioral functioning [3].

Despite being currently considered as two separate disorders, autism and schizophrenia present numerous overlaps in terms of risk and genetic factors, neurobiological characteristics, brain architecture, and clinical features [4], so much so that it is often difficult to differentiate between the core symptoms of one condition and those of the other. They show partially overlapping impairment in neurocognitive performance of social cognition, similar functional connectivity abnormalities in large-scale brain networks, and some shared neuroanatomical findings [5].

The clearest symptomatological overlap appears to be between the social communication deficits of autism spectrum disorder and the ‘negative symptoms’ (diminished or absent emotional expression, ‘avolition’, schizophrenic concretism, isolation, blunted affect) of the schizophrenia spectrum [6]. Other overlapping areas that, like negative symptoms, are related to the schizophrenia spectrum but are not part of the diagnostic criteria include mentalization and neurocognitive functioning [7]. Undoubtedly, more in-depth research on the impairment of the areas characterizing these two conditions could better define the differences between the symptomatological frameworks; however, even in this case there are potential overlapping symptoms. Specifically, taking into consideration the “positive symptoms” of psychosis, while hallucinations and delusions are more specific to the schizophrenic spectrum than to autism, the thought disorder and the impairment of the relational areas may be confused with the altered communicative aspects, especially the socio-pragmatic aspects of language, of autism [8]. In the latter, therefore, it is difficult to distinguish a psychotic thought disorder from an expressive and communicative deficit typical of the autism spectrum, or identifying the potential coexistence of both.

Despite these broad overlaps, key differences may facilitate clinicians in the differential diagnosis and identification of possible comorbidity between the two spectrums. First of all, the clinical course (onset, evolution, functioning over time) is crucial in differentiating the two conditions: whereas autism has a very early symptom onset, with many of the core deficits often already evident between 12 and 24 months of age and tending to remain more or less stable during childhood, the first symptoms of schizophrenic disorders usually develop from adolescence to young/middle adulthood and represent a clear change from their baseline [5,9]. The frequency and time interval of symptoms can also provide important indications. Psychotic symptoms are often episodic, transient, and short-lived, in contrast to autistic symptoms, which are typically stable and persistent throughout the individual’s development [8]. As far as individual functioning is concerned, in the case of autism, functional impairment is stable and present from childhood; in contrast, in schizophrenia spectrum disorders there is a significant deterioration in global functioning compared to the individual’s usual functioning [1].

Another main distinction between the two spectra exists in the linguistic/communicative domain, because language deficits can be detected in a subject with autism from the earliest stages of development, while thought disorganization in subjects who develop a psychotic condition tends to occur ex abrupto [1].

Even regarding delusional ideas, the cardinal symptoms of psychosis, one must be cautious in their interpretation, as atypical beliefs and idiosyncratic obsessions are very common in autism, and autistic preoccupations are often difficult to distinguish from delusional thinking in these subjects [10]. In cases where these develop during childhood alongside other neurodevelopmental deficits and are consistent with the subject’s restricted interests, without significant progression or worsening over time, a condition exclusive to the autism spectrum may be suspected [8]. Disperceptions (auditory, visual, or sensory), which represent a core symptom of the schizophrenic spectrum, are probably more difficult to differentiate from the sensory perceptual and self-talk anomalies typical of autism [11]; however, even in this case, the timing of onset, as well as the lack of delusional and external attributions of sensory symptoms, may point towards a diagnosis of autism without schizophrenic comorbidity [8,12]. Considering the “negative symptoms”, the lack of reciprocity in autism could be confused with the emotional and affective flattening of psychosis, but an inappropriateness of reciprocity is purely typical of autism [8]. Regarding the restricted and repetitive behaviors, a core feature of autism, although they may also occur in individuals in the schizophrenic spectrum, given the frequent comorbidity with obsessive compulsive disorder, they usually appear later, are less stable, and often correlate with delusional thoughts in these subjects [8,13]. Clinicians dealing with these conditions, especially developmental patients, need to have clear overlaps and differences to make an accurate differential diagnosis and intercept comorbid situations between the two spectra in time [1].

There is also frequent comorbidity between these two spectra, with calculated rates ranging from less than 1% to 60% [14,15]. Individuals with autism spectrum (AS) may, over the years, develop psychotic onset in comorbidity. Evidence is accumulating that individuals with AS are at greater risk of developing psychotic illnesses than the general population [16,17,18].

A recent meta-analysis shows that schizophrenia and bipolar disorder have substantially higher prevalence in adults with autism than estimates for the general population. The prevalence of AS among patients with a psychotic onset is 12 times higher than in the general population [19].

People with AS can have psychosis called “atypical”, that is with a more acute, transient course than that seen in the general population [20] Patients diagnosed with AS show schizophrenia spectrum traits in adolescence, and the behavioral overlap appears to go beyond negative schizotypal symptoms and also involves disorganized and positive symptoms [21]. Comorbidity has been observed between autism and prodromal conditions of psychosis [22]. With “prodromal psychosis”, we mean the period of subclinical signs and symptoms that precedes the onset of full-blown psychosis [23]. According to data from the North American Prodrome Longitudinal Study, 2.6% of Clinical High Risk (CHR) youth have a comorbid neurodevelopmental disorder [24]. Moreover, it has been estimated that approximately 2.6% of individuals identified as at risk for psychosis have comorbid neurodevelopmental disorders, which are important to identify as they predict greater severity of psychosis, more impaired functioning, and lower quality of life in the case of conversion to psychotic onset [25], with consequent worse response to initial pharmacological strategies [26]. The difficulty in formulating a differential diagnosis between the two spectra lies, therefore, both in the overlap of the respective clinical manifestations and in the possible incorrect interpretation of the symptoms by clinicians, who can mistake core symptoms of autism for symptoms of a psychotic nature and vice versa [9].

Misinterpretations of symptoms are even more common when faced with first psychotic episodes or, even earlier, with prodromal syndromes, compared to cases of full-blown psychosis [1].

Research on the identification of risk syndromes in populations of subjects with autism is still in its infancy; however, it is essential to identify prodromal symptoms also in these groups, given the evidence of worse outcomes in the case of coexistence between autism and psychotic risk, particularly with a greater impact of social functioning [6,27,28].

Just as the differential diagnosis between autism and schizophrenia is complex, differentiating between prodromal symptoms of psychosis and autistic symptoms also represents a real challenge, given the numerous overlaps, and this is even more so during adolescence [8].

At present, two approaches are used to assess psychotic risk: the ultra-high risk (UHR) [29] and the basic symptom approach [30,31]. The UHR criteria comprise the attenuated positive symptom prodromal syndrome (APS), the prodromal syndrome of brief, intermittent, limited psychotic symptoms (BLIPS), and the prodromal syndrome of genetic risk and impairment (GRDS) [32]. Two instruments investigate these risk syndromes: the Comprehensive Assessment of At-Risk Mental States (CAARMS) and the Structured Interview for Prodromal Syndromes (SIPS). Basic symptom criteria were defined by the presence of basic cognitive/perceptual (COPER) and/or cognitive symptoms (COGDIS). The European guide on early diagnosis suggests the use of UHR criteria and basic symptoms to identify the risk of psychosis [33].

Currently, the assessment of attenuated psychotic symptoms in patients with autism is conducted using the same tools available for non-autistic patients, with numerous clinical repercussions [8].

Even the tools currently considered the gold standard for the diagnosis of autism spectrum disorder, that is, the Autism Diagnostic Observation Schedule, Second Edition—ADOS-2 [34] and the Autism Diagnostic Interview, Revised—ADI-R [35], are not suitable for differentiating between common features of autism and psychosis [36,37]. The first one [34] is a standardized and semi-structured assessment of communication, social interaction, play, and imaginative use of materials for individuals with autism spectrum disorders, whose activities, directed to the child/youth, are related to communication, social interaction, imagination and creativity, restrictive and repetitive behaviors/interests, and other abnormal behaviors. Instead, the ADI-R [35] is a structured interview for parents that focuses on the systematic and standardized observation of behaviors mainly on four areas of functioning: Language and Communication, Reciprocal Social Interaction, Stereotypical Behaviors, and Restricted Interests.

A main issue of the interviews available for the assessment of psychotic risk certainly lies in the high linguistic load, which determines the greater difficulty in completing the interview by patients with autism and a higher rate of errors compared to neurotypical peers due to neurocognitive and communication deficits [38].

The present study aims to evaluate the presence of subthreshold psychotic symptoms and/or defined psychotic risk syndromes in children and adolescents with a diagnosis of autism spectrum disorder. Secondarily, we will compare the prevalence, type, and severity of psychotic risk symptoms in the sample with those of a group of patients at clinical high risk of psychosis (CHR-P) but without a diagnosis of autism spectrum disorder. It is hypothesized that there is some overlap between the two spectra, particularly regarding negative symptoms.

The purpose is to identify which symptoms overlap and differ most between the two conditions when comorbidity is not present. Moreover, given the frequent comorbidity described between the two spectrums, it is reasonable to expect that several patients with autism spectrum disorder will have subthreshold psychotic symptoms in their clinical history.

## 2. Materials and Methods

### 2.1. Participants

For the present study, 23 patients diagnosed with autism spectrum disorder and 14 clinical high-risk for psychosis (CHR-P) patients without autism were recruited in the period between March 2021 and September 2023 at the Child Neuropsychiatry Unit of the Department of Neuroscience and Mental Health of Sapienza University of Rome. Specifically, patients were enrolled at the Neurodevelopmental Disorders Service, the Adolescent Psychiatric Day Hospital, the Adolescent Psychiatric Inpatient Department, and the Adolescent Psychiatric Emergencies Department of the Unit.

Inclusion criteria for the group of patients with autism were: (a) an age between 8 and 17 years; (b) a diagnosis of autism spectrum disorder formulated or confirmed at our service according to the DSM-5 criteria [33] using the Autism Diagnostic Observation Schedule, Second Edition (ADOS-2) [34] for patients and Autism Diagnostic Interview, Revised (ADI-R) [35] administered to parents in absence of the patient; (c) an average intellectual functioning (full-scale IQ ≥ 70) measured through the Wechsler Intelligence Scale for Children, 4th edition (WISC-IV) or the Wechsler Adult Intelligence Scale, 4th edition (WAIS-IV) according to age; (d) knowledge of the Italian language such as to allow a reliable evaluation.

The exclusion criteria for the sample of patients with autism were any clinically significant medical condition that could affect the results of the study (for example, psycho-organic syndromes, epileptic syndromes, metabolic disorders, recent head injury) or the patient’s ability to take part in the study, aggressive or dangerous behavior such as to require immediate containment or instant pharmacological intervention or rated by a score of 6 on item G4 (“dysphoric mood”) of the SIPS scale, a current diagnosis of substance dependence established through the Diagnostic Interview for the Evaluation of Psychopathological Disorders in Children and Adolescents (K-SADS-PL DSM-5) [39] administered to the parents.

The CHR-P patient group included subjects aged between 8 and 17 years, with a definite diagnosis of at-risk mental state according to the Structured Interview for Prodromal Syndromes (SIPS) [29] and cognitive disturbances (COGDIS) criteria from the Schizophrenia Proneness Instrument for Children and Youth (SPI-CY), an average intellectual functioning (IQ ≥ 70) measured through the WISC-IV or the WAIS-IV according to age, and knowledge of the Italian language such as to allow a reliable evaluation. The exclusion criteria for this group were the same as the autism group, but, in addition, they should not have a diagnosis of autism spectrum disorder.

For both groups, in the case of patients with borderline intellectual functioning (IQ between 70 and 85), eligibility for the study was assessed on a case-by-case basis, depending on cognitive, linguistic, metalinguistic, and self-reflective skills, assessed through WISC/WAIS and clinical non-structured interviews.

During the evaluation, a concomitant psychotic onset was detected in three of the autistic patients of the autistic sample, so they were considered a separate population and excluded from the statistical analysis due to the number being too limited to allow the creation of a third group.

Participation in the research was subject to the signing of informed consent by the parents of the minors in both samples, based on the ICH-Good Clinical Practice guidelines [40].

### 2.2. Procedure

The evaluation of the patients enrolled in both samples, with their respective parents or legal guardians, was conducted by three medical doctors specialized in child and adolescent Neuropsychiatry and by a medical doctor specialized in Psychiatry with experience in neurodevelopmental and psychopathological disorders of developmental age relating to the Department of Human Neurosciences, Division of Child Neuropsychiatry.

In the first step, socio-demographic information, medical, developmental and family history were collected, and psychometric assessment by self-report tests was carried out for clinical purposes and the present research. Also, social and role functioning was assessed.

During the subsequent visits, the clinical diagnosis of subjects was made through the administration of the Diagnostic Interview for the Evaluation of Psychopathological Disorders in Children and Adolescents—K-SADS-PL-5 [39] according to the criteria of the Diagnostic and Statistical Manual of Mental Disorders, 5th edition—DSM-5 [33]. The interview was administered both to the patient and the parents. The discussion between the investigators, who conducted the interviews, and the clinicians of the team, who were caring for the patient, resulted in a final clinical diagnosis.

The subjects of both samples underwent a textual evaluation of cognitive functioning using multicomponent tools, that is, the Wechsler Intelligence Scale for Children, 4th edition (WISC-IV) [41], for patients up to 16 years of age, and the Wechsler Adult Intelligence Scale, 4th edition (WAIS-IV) [42], for patients aged 16 and over, to calculate the intelligence quotient (IQ) and thus determine eligibility for the study.

The clinical diagnosis of CHR-P was established through the gold standard for the detection of psychotic risk, that is, the combination of the SIPS and COGDIS (SPI-CY) criteria, currently validated for the definition of an at-risk mental state for psychosis. CHR-P participants had to meet at least one of the following criteria: (1) attenuated positive symptoms (APS), (2) brief, limited, or intermittent psychotic symptoms (BLIPS), (3) genetic risk for psychosis, with a deterioration in functioning in the past year (GRDS), and (4) two or more of nine cognitive basic symptoms (COGDIS).

In the end, the subjects of both groups and their respective parents were administered the two tools currently considered the gold standard for the diagnosis of autism spectrum disorder in order to evaluate the presence and severity of autistic symptoms: the Autism Diagnostic Observation Schedule, Second Edition (ADOS-2) [34] to patients and the Autism Diagnostic Interview, Revised (ADI-R) [35] to parents. The entire assessment required 5 visits. The interviewers (EM, VM, AM, LT) were trained for administering SIPS and SPI-CY under the supervision of Prof. Josef Parnas, Prof. Andrea Raballo, and Prof. Frauke Schultze-Lutter (EM) and Prof. Andrea Raballo and Dr. Eva Gebhardt (VM, AM, LT).

### 2.3. Assessment Tools

The K-SADS-PL for DSM-5 is a semi-structured diagnostic interview that ascertains both lifetime and current diagnostic episodes in children and adolescents aged 6 to 18 years according to DSM-5 criteria. The tool can generate 35 child and adolescent psychiatric diagnoses, with the addition of five modules for mood dysregulation disorder (DMDD), avoidant restrictive food intake disorder (ARFID), selective mutism, uncontrolled eating disorder, and autism spectrum disorder [33].

The ADOS-2 [43] is a standardized and semi-structured assessment of communication, social interaction, play, and imaginative use of materials for individuals with autism spectrum disorders. The activities directed to the child/youth fall within five categories: Language and Communication, Reciprocal Social Interaction, Imagination and Creativity, Stereotypical Behaviors and Restricted Interests, and Other Abnormal Behaviors. The scores are instead organized into two main areas: Social Affect (SA), which includes Communication and Reciprocal Social Interaction, and Restricted and Repetitive Behavior (RRB). The ADOS-2 test has 5 modules organized according to language skills and patient age. Patients aged 8 to 17 are tested with modules 3 and 4 of the ADOS-2 scale. Since the validation of the instrument, module 3 includes a standardized calibrated severity score (CSS) from 1 to 10 based on the total raw score. Module 4 of the current ADOS-2, however, does not include a CSS and provides only a raw score, which is a less reliable measure of autism severity and more influenced by individual characteristics. In the present study, however, a CSS was also obtained for the scores of module 4 by referring to the calculation table proposed by the Lord’s team in 2014 [43].

The ADI-R [35] is an interview designed to be used in combination with the Autism Diagnostic Observation Schedule-2 (ADOS-2); it collects descriptions of a subject’s behavior throughout his life so that it is possible to determine whether his developmental path and behavior characteristics meet the criteria for the diagnosis of autism spectrum disorder. The ADI-R is aimed at parents or educators of subjects from early childhood to adulthood, with a mental age above 2 years. It focuses on the systematic and standardized observation of behaviors rarely found in non-clinical subjects and mainly on four areas of functioning: Language and Communication, Reciprocal Social Interaction, Stereotypical Behaviors, and Restricted Interests. It consists of an interview protocol and five algorithms that can be used at various ages for diagnosis and intervention. The interview includes 93 items divided into eight sections, three of which are important for detecting autism spectrum disorder: language and communication functioning, social development, and play, interests, and behaviors. The ADI-R algorithms are modules on which the coding of the fundamental items (up to 42) must be reported and systematically combined to produce formal and interpretable results. A subject is assessed through a single module, depending on whether this is needed for diagnosis or surgery and depending on his age. One of two diagnostic algorithms (2.0–3.11 years/4.0 years and older) is used to make the formal diagnosis and arrive at an overall estimate of the severity of the disorder.

The Structured Interview for Prodromal Syndromes/Scale of Prodromal Symptoms (SIPS/SOPS) [29] is a structured interview that allows us to identify the presence of Ultra-High-Risk (UHR) criteria and the three different prodromal syndromes (APS, BLIPS, or GRDS). The scale is characterized by 19 items divided into four domains: positive, negative, disorganization, and general symptoms. It also involves the identification of the Criteria for Schizotypal Personality Disorder, as well as the evaluation of functioning through the Global Assessment of Functioning (GAF) [44]. Each positive symptom is assigned a score on a Likert scale from 0 (not present) to 6 (severe and psychotic). The APS is characterized by at least one of the positive items with a score between 3 and 5. This item has to begin within the past year or has to increase its severity by almost one or more scale points in the last year. The frequency does not correspond to an onset.

BLIPS is diagnosed if a positive item obtains a score of 6 (severe and psychotic), and this symptom has to have enriched the psychotic level in the past three months. Moreover, these symptoms must be present for at least several minutes daily, at least once a month. A positive item is evaluated as present when its score is equal to between 3 and 6. Calculating the total score of the five positive items out of all scores (0–6) makes it possible to estimate the severity of the UHR symptom criteria.

The COPER and COGDIS criteria are taken from the semi-structured Schizophrenia Proneness Interview Instrument—Child and Youth version (SPI-CY) [45] designed to detect and evaluate basic symptoms in children from 8 years of age and adolescents. The COPER (cognitive/perceptive) criteria are more sensitive, while the COGDIS (cognitive) criteria are more specific; the latter represent criteria for basic symptoms suggested as complementary to the UHR criteria for early diagnosis of psychosis. The severity of underlying symptoms is rated based on their onset frequency on a Likert scale ranging from 0 (not present) to 6 (present daily). A basic symptom is considered clinically present if its score is between 3 and 6.

The Adaptive Behaviour Assessment System, Second Edition (ABAS-II) [46] was completed by the parents of all the subjects in both samples. This is a self-administered questionnaire to evaluate their adaptive functioning that measures daily living skills, i.e., what people can do without the help of others. It can detect these abilities in subjects aged between 0 and 89 who have pervasive developmental disorders, intellectual disabilities, neuropsychological problems, dementia, learning difficulties, biological risk factors, and sensory or physical impairments. The tool investigates 10 adaptive areas, attributable to the 3 conceptual, social, and practical domains, to which the Motor skills area is added, limited to the evaluation of children aged 0 to 5 years.

The Global Functioning:Social (GF:S) and Global Functioning:Role (GF:R) scales [47,48] are two interviews that assess social and role functioning. GF:S and GF:R scores range from 1 (extreme dysfunction) to 10 (highest functioning). Each scale has 3 different scores: current functioning, i.e., lowest level of functioning in the last month, lowest level, and highest in the last year.

The GF:S assesses the quantity and quality of peer relationships. In addition, the scale assesses the conflicting nature of relationships, the presence of age-appropriate affective relationships, and interest in them. Finally, it also investigates the quantity and quality of family relationships. Particular attention is given to relationship avoidance and social withdrawal.

The GF:R assesses performance at school, work, or home. The scale assesses the individual’s level of independence in these activities and/or the need and level of support required for acceptable functioning.

Both scales assess functioning independently of the etiology of social dysfunction and clinical symptomatology.

The Global Assessment of Functioning (GAF) scale, included in the SIPS/SOPS, assesses current and highest global functioning over the past year [44].

The score ranges from 0 (extreme dysfunction) to 100 (highest functioning). The subject’s etiology and symptomatology also impact the GAF scores.

### 2.4. Data Analysis

The comparison analyses between the groups (autism and CHR) were performed by reporting the distributions of the nominal variables (frequencies) in two-dimensional contingency tables and then were studied by applying the chi-square test.

Continuous variables were described through mean, median, standard deviation, and confidence interval. Continuous variables were tested with a one-way analysis of variance (Student’s *t*-test for pairwise comparisons) in case of normal distribution and Mann–Whitney U test in case of non-normal distribution.

The correlations between numerical variables were studied with the Spearman rho correlation coefficient, a non-parametric test whose choice is dictated by the notable differences between the variability ranges of the variables involved in the analysis. The correlation analysis was performed for continuous variables that suggested possible mutual influences of clinical significance. For the key findings, we have calculated the effect sizes.

The null hypothesis adopted (Ho) was defined as the absence of differences between the distributions being compared and the absence of correlation/concordance between variables. The reading of the results and the discussion of the results of the applied tests was performed using a value of alpha = 0.05 to reject the null hypothesis.

IBM SPSS statistical analysis software in version 27.0 was used for data analysis [49].

## 3. Results

### 3.1. Sociodemographic Characteristics and Intelligence Quotient

The sample examined for the statistical analysis consists of 34 patients. Three of the patients with autism spectrum disorder enrolled were excluded from the analysis, as they had an ongoing psychotic onset, and their number was too small to create a third comparison group. Therefore, the two analyzed samples comprise 20 patients with autism and 14 CHR patients without autism.

The characteristics of the sample are reported in Table 1 and Table 2. The 34 patients range in age from 8 to 17 years (mean age: 13.94, with SD = 2.75). The average age of the subjects with autism is 12.85, and that of the CHR patients is 15.50, with a statistically significant difference between the two groups (*p* = 0.004).

The total sample consists of 20 males and 14 females, with a percentage of males of 58.8%. Considering the sex distribution in the two samples, 80% of patients with autism are male; in contrast, 71.5% of CHR patients without autism are female, with a difference between the two groups that is statistically significant (*p* = 0.003).

An independent-sample *t*-test was run to determine if there were differences in engagement to an advertisement between males and females. The data had no outliers, as assessed by inspection of a boxplot. Engagement scores for each level of gender were normally distributed, as assessed by Shapiro–Wilk’s test (*p* > 0.05), and variances were homogeneous, as assessed by Levene’s test for equality of variances (*p* = 0.174). The advertisement was more engaging to male viewers (M = 5.56, SD = 0.35) than female viewers (M = 5.30, SD = 0.35), with a statistically significant difference, M = 0.26, 95%CI [0.04, 0.48], t(38) = 2.365, *p* = 0.023, d = 0.75.

Both groups present similar full-IQ mean values (Table 3) with a non-significant difference.

### 3.2. Psychopathological Evaluation

The psychopathological dimensions of SIPS (total, positive, negative, disorganization, and general symptoms) were therefore evaluated and compared in the group of patients with autism and the CHR group. The means, standard deviations, and levels of statistical significance are reported in Table 4. The mean SIPS total scores were 25.20 (SD = 14.4) in patients with autism and 44.71 (SD = 14.1) in CHR patients, with a statistically significant difference between the two groups (*p* = 0.002). Comparing each of the four dimensions of the SIPS in the two independent groups with the Mann–Whitney U test, positive symptoms were significantly higher in CHR patients (*p* < 0.001), as were negative symptoms (*p* = 0.012), symptoms of disorganization (*p* = 0.030), and general symptoms (*p* = 0.011).

None of the patients with autism in the sample were found to be at psychotic risk due to failure to reach the SIPS cut-off, so a comparison was made between symptoms below the clinical threshold for symptoms of psychotic risk.

Therefore, the individual items of the SIPS were analyzed, the means, standard deviations, and significance levels of which are reported in Table 5.

#### 3.2.1. Differences in the Positive Symptoms of SIPS

As regards the positive symptoms of SIPS, the symptoms relating to “Unusual content of thought/Delusional ideas” (*p* < 0.001), “Suspiciousness/Ideas of persecution” (*p* < 0.001), and “Misperceptions/Hallucinations” (*p* < 0.001) were significantly more present in the UHR sample than in the sample with autism. There was no statistically significant difference between the two groups regarding “Ideas of Greatness” (*p* < 0.486) and “Disorganized Language” (*p* = 0.342).

#### 3.2.2. Differences in the Negative Symptoms of SIPS

Among the negative symptoms of SIPS, significant differences emerged between the two groups regarding “Loss of willpower” (*p* = 0.002), “Perception of self-emotions” (*p* = 0.002), and “Occupational functioning” (*p* < 0.001), with all symptoms significantly more present in the CHR group. Compared to all the negative symptoms, only the symptoms of “Ideal Richness” were more represented in the group of patients with autism, but with a non-statistically significant difference in the comparison with the CHR group (*p* = 0.066). The highest significance is highlighted for the “Occupational functioning” negative symptoms.

#### 3.2.3. Differences in the Symptoms of Disorganization of SIPS

As regards the symptoms of disorganization, only the symptoms relating to “Bizarre Ideation” (*p* = 0.045) and “Attention and concentration disorders” (*p* = 0.017) were statistically more represented in the CHR group; in contrast, the symptoms of “Strange behavior or appearance” (*p* = 0.628) and “Impairment of personal hygiene” (*p* = 0.384) were equally higher in the CHR group but with a non-statistically significant difference.

#### 3.2.4. Differences in the General Symptoms of SIPS

Among the general symptoms, the symptoms “Dysphoric mood” (*p* = 0.032) and “Reduced tolerance to normal stress” were statistically higher in the group of CHR patients (*p* = 0.005); in contrast, the symptoms of “Sleep disorders” and “Motor disorders” were more represented, the former in CHR and the latter in patients with autism, but without statistically significant differences between the two groups (*p* = 0.085 and *p* = 0.292, respectively).

#### 3.2.5. COPER and COGDIS Criteria

An analysis was also conducted on the presence of the COPER and COGDIS risk criteria of the SPI-CY in the two groups (Table 6). Both criteria were more present in CHR subjects, while only 2/20 patients with autism (10.0%) presented COPER and COGDIS risk criteria. The difference in the two groups was statistically significant (*p* < 0.001 for both criteria).

### 3.3. Autistic Symptoms

Analysis and comparisons were conducted on the four areas of the ADI-R (A = Reciprocal social interaction; B = Qualitative anomalies in communication; C = Restricted, repetitive and stereotyped behavior patterns; D = Developmental anomalies evident at/before the age of 36 months) in the two groups, taking into consideration the clinical cut-off of the respective areas (Table 7) and without considering these cut-offs (Table 8). There was one missing in the CHR patient group, so the results of 13/14 patients were considered. Based on whether the clinical cut-offs were exceeded or not, a greater prevalence of symptoms above the clinical threshold was highlighted in patients with autism in the areas of “Reciprocal social interaction” (*p* = 0.002), “Qualitative anomalies in communication” (*p* < 0.001), “Restricted, repetitive and stereotyped patterns of behavior” (*p* < 0.001), and “Developmental anomalies evident at/before 36 months” (*p* = 0.003), with a statistically significant difference between the two groups in all four areas.

Considering the scores of the two groups in the four areas of the ADI-R without considering the clinical cut-offs, the distribution of the symptoms investigated in the two groups was evaluated, and the means, standard deviations, and statistical significance of which are reported in Table 8. For all four areas, higher scores were found in patients with autism compared to the CHR group, with a statistically significant difference, but to a greater extent for areas B and D (*p* = 0.001).

A correlation analysis was then conducted between the negative symptoms of SIPS and area A, Reciprocal social interaction, of the ADI-R since reports in the literature have shown a frequent overlap between these two symptomatological domains. There was no statistically significant correlation between the total negative symptoms of SIPS and the total area A of the ADI-R (two-tailed sign. = 0.374).

### 3.4. Global Functioning

An analysis was conducted on the GAF scale, considering the scores relating to the current moment, the lowest in the past year, and the highest in the past year in the two groups (Table 9). Current functioning was significantly lower in the CHR group (*p* = 0.008), as was lower functioning in the past year (*p* = 0.001).

Analyses and comparisons were conducted of the three domains and the total of the ABAS-II (GAC = total; DAC = conceptual domain; DAS = social domain; DAP = practical domain) in the two groups, taking into consideration the clinical cut-off of the respective domains (Table 10) and without considering these cut-offs (Table 11). There was one missing in the group of UHR patients, so the results of 13/14 subjects were analyzed. Considering a composite score of less than 70 as the clinical cut-off, statistically significant differences were found between the two groups in the conceptual (*p* = 0.050) and social (*p* = 0.006) domains. Clinically significant scores for both domains were preponderant in the autism sample.

The analysis conducted on the scores of the three domains and the scale’s total without considering the clinical cut-off highlighted a statistically significant difference in the social domain (*p* = 0.002), with lower scores, and therefore lower social functioning, in the patient group with autism.

## 4. Discussion

The study explores the psychopathological dimensions of psychotic risk, autistic symptomatology, and functioning in two samples of children and adolescents with autism and clinical high risk, respectively.

The average age of patients with autism was significantly lower than the average age of CHR subjects, probably because of the two diagnoses, as patients belonging to the neurodevelopmental disorders service more often access assessment for autism at earlier ages; in contrast, those belonging to psychiatric services tend to show symptoms of psychotic risk at a later age. This finding is in line with the evidence present in the literature: while the onset of autistic symptoms is very early (sometimes as early as 12–24 months of age), the prodromal symptoms and the first psychotic symptoms usually develop between adolescence and young/middle adulthood [9,50].

Analyzing the sex distribution in the two samples, an undoubted prevalence of male subjects emerges among patients with autism and an undoubted prevalence of female subjects in the CHR group. The greater prevalence of autism spectrum disorder in males is a fact also supported by the literature [13,51,52]. On the contrary, the prevalence of females in the UHR sample does not agree with the available evidence, which suggests a greater presence and severity of prodromal symptoms in males, especially regarding negative symptoms and social functioning [53,54].

In our autism sample, none of the subjects appeared to present a condition of psychotic risk, while the three eliminated from the analysis directly presented psychosis that had already begun. In the literature, on the contrary, a prevalence of UHR symptoms in subjects with autism is described, which can reach up to 78.0% [27,38,45]. This could be attributed to a masking effect of prodromal symptoms by some autistic symptoms (especially relating to communication, social interaction, and behavior), which would bring these subjects to clinical attention for psychiatric symptoms only after the prodromal phase when the onset is already underway. This would underline even more the importance of a priori psychotic risk screening in these populations, as they are more at risk of presenting a schizophrenia spectrum disorder compared to the general population and neurotypical clinical populations [8]. Furthermore, we could hypothesize a different mechanism for the onset of psychosis in neurodiverse individuals compared to neurotypical individuals, with consequent differences in the evolutionary process of the disease. Psychosis in autism could be seen, from this point of view, a direct derivative of neurodiversity rather than a disease by itself. However, further studies are needed to investigate this hypothesis.

Given that patients with autism were not at risk of psychosis due to failure to reach the clinical threshold for risk symptoms, potentially premorbid symptoms, i.e., even earlier than prodromal symptoms, were analyzed. While positive symptoms were more specific to risk conditions, negative, disorganization, and general symptoms did not differ significantly between the two groups. This also reflects the greater difficulty in the differential diagnosis of these three symptom areas [1,8].

Considering the individual items of the four psychopathological dimensions of the SIPS, the items in the positive symptoms that are best able to discriminate between a psychotic risk condition and a non-risk autism spectrum appear to be those relating to the unusual content of thought and delusional ideas, suspiciousness/persecution, and dysperceptive symptoms.

Among the negative symptoms, no significant differences emerged in the items concerning social anhedonia, the expression of emotions, and ideational richness, with the latter, however, being more affected in autism. At the same time, significance was present in the items relating to the perception of emotions and occupational functioning. This could be due to an effect inherent in the very structure of the interview questions: while the items in which no significant differences emerged do not include a temporal criterion that indicates a change compared to the previous situation, the items on emotion perception and occupational functioning to obtain a score higher than 1 imply that there has been a clear change compared to the past. The literature confirms the data regarding the greater stability of autistic symptoms over time compared to prodromal symptoms, for which a fracture line between the previous and current status is reported [5,9]. Interestingly, the two groups differed in the “experience of emotions and self”. This item assesses symptoms attributable to a deficit of primary intersubjectivity and an alteration of the basic self, such as loss of sense of self, feeling profoundly changed, strange or unreal, and a sense of distance when talking to others and others. This would lead us to think that the primary intersubjectivity and the sphere of the self are more specifically altered in a condition of psychotic risk rather than in autism and that, therefore, their investigation could represent a clinically valid supplement in the differential diagnosis between autism and the schizophrenia spectrum [55]. These anomalies of the self, which are more frequent in CHR-P subjects than in autistic subjects, could underlie and explain the differences found at the level of positive symptoms.

Nevertheless, other studies have suggested the presence of a disorder of self-awareness underlying both autism and schizophrenia [56], so further studies are needed to corroborate one or the other hypothesis.

It should be underlined again that the low number does not make these results generalizable; therefore, an expansion of the sample is necessary.

From the analysis of global functioning in the two groups, the datum that stands out most is the notable reduction in functioning over the year in the autism sample. The literature confirms a greater decline in the global functioning of UHR subjects over time, while functioning in the autism spectrum would generally remain unchanged [1]. One explanation for this conflicting finding may lie in the recent appearance (within 12 months prior to the evaluation) of psychiatric comorbidities in all autistic subjects evaluated, which may have contributed to the worsening of functioning more than the autistic symptomatology itself [57].

For the analysis of autistic symptoms, we chose to take into consideration exclusively the scores recorded on the ADI-R and not those on the ADOS-2. This decision is to be attributed first to the differences in the items necessary for calculating the final score of the two modules used (*module 3* up to pre-adolescence and *module 4* from adolescence onwards). Although a comparison score is available, which should make the two modules comparable, these scores result from calculations that, in the first phase, involve the inclusion of scores relating to restricted and repetitive behaviors. At the same time, in the second phase, they do not. Furthermore, the literature data confirm the poor specificity of modules 3 and 4 of the ADOS-2 in identifying autism spectrum situations in children and adolescents with high cognitive functioning and in disconfirming the diagnosis in neurotypical patients with concomitant psychiatric problems [58,59,60]. Therefore, it was decided to use the tool exclusively to formulate/confirm the autism diagnosis with the ADI-R interview, as per clinical practice [61].

In all areas of the ADI-R, considering the clinical cut-offs and considering only the presence and severity of symptoms, a statistically significant difference was found between the two groups, confirming the discriminative capacity of the interview between autism spectrum and other conditions [61]. We wanted to compare the total negative symptoms of the SIPS and the symptoms of area A (reciprocal social interaction) of the ADI-R to investigate a possible correlation. It turned out that the two symptom areas do not seem to be related and, therefore, could be considered different. Therefore, the two SIPS and ADI-R tools would be able to discriminate well between these two symptom domains, which would be more specific to the two conditions than previously thought. Previous studies had suggested a greater specificity of the positive symptoms of schizophrenia and, on the contrary, an important overlap between negative symptoms and atypia in social behavior in the schizophrenia spectrum and autism [6,62]. This overlap has been explained mainly through similar neurobiological mechanisms investigated through functional imaging studies conducted during social cognition tests [63]. However, there is more recent literature data, according to which there is a clear difference between the two domains, as a poverty of reciprocity is inherent in the negative symptoms of the schizophrenic spectrum. At the same time, in the social interaction deficits of autism, it is instead a question of inappropriateness of reciprocity, with the latter particular to the autistic spectrum [8]. Further studies conducted on larger samples, and which individually analyze the symptoms that make up the two domains, could confirm these findings and lead to a reevaluation of the role of negative symptoms, which are currently not included among the essential criteria for making a diagnosis [64], in the etiopathogenesis of schizophrenia.

The study has some limitations to consider. First, the small size of the two samples and the lack of a clinical control group without autism and UHR and a healthy, non-help-seeking control group limits the generalizability of the results. The study comprises help-seeking symptomatic patients referred to third-level diagnostic units; therefore, sample preselection bias cannot be ruled out. The low number is mainly attributable to time and staff training costs, as the four interviews included in the evaluation require specific certified training, and their administration requires a prolonged amount of time, with the need for several meetings per patient and their parents. Furthermore, not all patients belonging to the services involved were eligible for enrollment due to cognitive functioning below the norm or within the lower limits of the norm but with poor language skills, language barrier in foreign patients, parents/patients not compliant with the assessment, and patients in psychiatric acute care not being evaluable. Another limitation to consider is the wide age range of the entire sample, which significantly impacts the analyses. However, this is a preliminary study, and increasing the size of the sample and the breadth of the age range could affect the results to a lesser extent.

In addition to the very wide age range, the study sees an imbalance of subjects with autism towards lower ages and of subjects with UHR without autism towards higher ages. This is attributable to the effect of the two diagnoses themselves, as patients more often access evaluation for autism spectrum disorder at an earlier age, and those who are evaluated at psychiatric services tend to show symptoms of psychotic risk not before preadolescence. This bias could be partially overcome by providing psychotic risk assessments on older autistic patients already diagnosed at preschool/school age at the facility.

It is certainly of great clinical and research interest to identify specific protocols that can intervene at the first signs of risk of thought disorganization in autistic individuals. It is also important to implement longitudinal studies that may evaluate vulnerability and resilience factors in populations of individuals with autism. Especially in autistic individuals with excellent outcomes and without psychiatric comorbidities, it is crucial to investigate, even through retrospective studies, the pathways that have favored their positive development.

## 5. Conclusions

This preliminary data highlighted significant differences in age, sex, involved symptom dimensions, and functioning of the subjects evaluated. The age imbalance in favor of earlier ages in autistic subjects and adolescents in UHR subjects reflects the course of the symptomatology of both conditions [5,9]. The gender distribution, which in the case of autism follows current evidence [13,51,52], while for UHR subjects is in contrast with the literature data [53,54], can be further investigated by expanding the size of the two samples and limiting the pre-selection biases of the subjects. Although none of the patients in our sample with autism were found to be at psychotic risk, many of them, not suspected of having psychotic or prodromal symptoms, still presented symptoms below the clinical threshold for risk, which could be considered premorbid, and three were identified with full-blown psychotic onset. Therefore, an a priori screening of psychotic risk in neurodiverse populations is fundamental to prevent more serious conditions, which can lead to a significant level of suffering and costs in social, therapeutic, and public health terms. Greater specificity was found for negative prodromal symptoms, particularly for those related to disorders of intersubjectivity and basic self. Greater specificity was found for negative prodromal symptoms, particularly those related to disturbances of intersubjectivity and basic self; this could suggest a way to improve the differential diagnosis between autism and the schizophrenic spectrum by investigating anomalies of experience. However, further studies are necessary to also investigate a possible alteration of intersubjectivity and awareness of the self in subjects with autism, considering the conflicting literature data [55,56]. Our data on autistic symptoms would confirm the greater discriminative capacity of the ADI-R diagnostic interview regarding these [61]. The non-significant correlation found between the area of social interaction in autism and negative symptoms in schizophrenia would strengthen the hypothesis of specificity of schizophrenic negative symptoms, contrary to what has been described in the past; it could suggest a reevaluation of the importance of negative symptoms in the diagnosis of schizophrenia. The difficulties in relating to others in individuals at risk of psychosis could arise from negative symptoms such as social anhedonia, avolition, anomalous self-experiences, and flattening of effectivity. So, they may differ from the anomalies of social interaction found in patients with autism. More data and an analysis of the individual symptoms of the two areas would be necessary to support this hypothesis further. Accordingly, we could hypothesize a different clinical course of psychotic conditions in subjects with and without autism spectrum disorder. In this view, the psychotic onset in an autistic subject could be seen as a consequence of neurodiversity and not as a mental pathology, as happens instead for the person suffering from schizophrenia.

However, further studies are needed to investigate the diversity in the mechanisms of onset of psychosis in neurotypical and neurodiverse individuals, respectively.

The emerging results prove to be particularly important in the life course of autistic individuals. Being able to detect early possible psychotic alterations in thought, which are often masked by the typical rigidities of autism, would allow for timely and more targeted intervention and certainly a better prognosis.

## Figures and Tables

**Table 1:** Sociodemographic characteristics of participants: gender.

	Total		AS		CHR-P		Sign
	*n*	%	*n*	%	*n*	%	
Gender							χ² = 8.993 **
Female	14	41.2	4	20	10	71.4	
Male	20	58.8	16	80	4	28.6	

** *p* ≤ 0.01. AS = autism spectrum; CHR-P = clinical high risk for psychosis.

**Table 2:** Sociodemographic characteristics of participants: age.

	Total		AS		CHR-P			
	*M* (SD)	95%CI	*M (SD)*	95%CI	*M (SD)*	95%CI	Sign	Cohen’s d
Age	13.94 (2.7)	12.19–14.85	12.85 (2.7)	11.5–14.15	15.50 (1.8)	14.42–16.58	F = 9.636 **	1.154

** *p* ≤ 0.01; AS = autism spectrum; CHR-P = clinical high risk for psychosis.

**Table 3:** Full IQ of participants.

	TOTAL		AS		CHR-P			
	*M* (SD)	95%CI	*M (SD)*	95%CI	*M (SD)*	95%CI	Sign	Cohen’s d
Full-IQ	105.52 (13.732)	100.26–110.4	105.71 (15.369)	97.80–113.61	105.20 (11.163)	97.21–113.19	F = 0.008	0.037

AS = autism spectrum; CHR-P = clinical high risk for psychosis; IQ = intelligence quotient.

**Table 4:** Means, standard deviations, and statistical differences of psychopathological dimensions.

Measure	AS		CHR-P		Mann-Whitney U Test	r
	*M (SD)*	95%CI	*M (SD)*	95%CI		
SIPS P	5.40 (4.5)	3.28–7.52	11.36 (3.2)	9.54–13.18	45.000 ***	0.574
SIPS N	10.30 (7.1)	6.98–13.62	16.71 (6.7)	12.84–20.59	68.500 *	0.431
SIPS D	3.25 (3.3)	1.72–4.78	5.93 (3.6)	3.86–8.00	78.500 *	0.374
SIPS G	6.25 (4.0)	4.36–8.14	10.00 (4.1)	7.64–12.36	67.500 *	0.44
SIPS TOTAL	25.20 (14.4)	18.45–31.95	44.71 (14.1)	36.55–52.88	51.000 **	0.586

* *p* ≤ 0.05; ** *p* ≤ 0.01; *** *p* ≤ 0.001. AS = autism spectrum; CHR-P = clinical high risk for psychosis.

**Table 5:** Means, standard deviations, and significance levels of the individual items of SIPS.

Measure	AS	CHR-P	Mann-Whitney U Test
	*M (SD)*	*M (SD)*	
SIPS P1	1.00 (1.0)	3.21 (0.9)	17.000 ***
SIPS P2	1.40 (1.3)	3.21 (1.4)	48.500 ***
SIPS P3	0.50 (1.0)	0.86 (1.5)	124.000
SIPS P4	1.10 (1.5)	3.29 (1.2)	36.500 ***
SIPS P5	1.45 (1.6)	0.93 (1.1)	115.000
SIPS N1	2.45 (1.8)	3.29 (1.7)	102.000
SIPS N2	1.30 (1.6)	3.50 (1.8)	54.000 **
SIPS N3	2.05 (1.9)	2.50 (1.6)	121.000
SIPS N4	1.45 (1.3)	3.14 (1.5)	54.000 **
SIPS N5	2.15 (1.6)	1.21 (1.2)	87.000
SIPS N6	0.90 (1.4)	3.21 (1.6)	40.500 ***
SIPS D1	0.85 (1.3)	1.00 (1.2)	127.500
SIPS D2	0.85 (0.9)	1.71 (1.3)	85.000 *
SIPS D3	1.10 (1.2)	2.29 (1.4)	74.000 *
SIPS D4	0.45 (0.9)	0.93 (1.5)	120.000
SIPS G1	1.65 (1.5)	2.71 (2.0)	92.000
SIPS G2	1.80 (1.6)	3.29 (2.1)	80.000 *
SIPS G3	1.30 (1.6)	0.86 (1.4)	112.000
SIPS G4	1.50 (1.7)	3.14 (1.4)	62.000 **

* *p* ≤ 0.05; ** *p* ≤ 0.01; *** *p* ≤ 0.001. AS = autism spectrum; CHR-P = clinical high risk for psychosis. P1 = Unusual thought content/Delusional ideas; P2 = Suspiciousness/Ideas of persecution”; P3 = Ideas of greatness; P4 = Misperceptions/Hallucinations; P5 = Disorganized Language. N1 = Social anhedonia; N2 = Loss of willpower; N3 = Expression of emotions; N4 = Perception of Self emotions; N5 = Ideational richness; N6 = Occupational functioning. D1 = Strange behavior or appearance; D2 = Bizarre Idea; D3 = Attention and concentration disorders; D4 = Compromise of personal hygiene. G1 = Sleep disorders; G2 = Dysphoric mood; G3 = Motor disorders; G4 = Reduced tolerance to normal stress.

**Table 6:** Presence of the COPER and COGDIS risk criteria of the SPI-CY in the two groups.

	AS		CHR-P		Sign
	*n*	%	*n*	%	χ²
COGDIS+	2/20	10.0	12/14	85.7	13.607 ***
COPER+	2/20	10.0	10/14	71.4	19.49 ***

*** *p* ≤ 0.001. AS = autism spectrum; CHR-P = clinical high risk for psychosis. COPER criteria = cognitive/perceptual; COGDIS criteria = cognitive.

**Table 7:** Analysis and comparisons among the four areas of the ADI-R (considering the clinical cut-off of the respective area).

	AS		CHR-P		Sign.
	*n*	%	*n*	%	*χ²*
ADI-R A (>cut-off)	14/20	70.0	2/13 (15.9)	15.4	9409 **
ADI-R B (>cut-off)	15/20	75	2/13 (16.1)	15.4	11,211 ***
ADI-R C (>cut-off)	15/20	75	2/13 (18.4)	15.4	11,211 ***
ADI-R D (>cut-off)	15/20	75	2/13	15.4	8567 **

** *p* ≤ 0.01; *** *p* ≤ 0.001. AS = autism spectrum; CHR-P = clinical high risk for psychosis. A = Reciprocal social interaction; B = Qualitative anomalies in communication; C = Restricted, repetitive and stereotyped behavior patterns; D = Developmental anomalies evident at/before 36 months.

**Table 8:** Analysis and comparisons among the four areas of the ADI-R (without considering the clinical cut-off of the respective area).

	AS		CHR-P		Sign.	r
	M (SD)	95%CI	*M (SD)*	95%CI	Mann-Whitney U Test	
ADI-R A	12.30 (6.5)	9.27–15.33	6.46 (4.6)	3.70–9.23	60.000 **	0.44
ADI-R B	10.70 (4.7)	10.70–8.51	4.38 (2.2)	5.07–5.70	35.500 ***	0.60
ADI-R C	4.50 (3.1)	3.05–5.95	1.77 (1.2)	1.06–2.47	64.000 *	0.42
ADI-R D	1.50 (1.2)	0.92–2.08	0.23 (0.4)	0.030.50	49.000 ***	0.54

* *p* ≤ 0.05; ** *p* ≤ 0.01; *** *p* ≤ 0.001. AS = autism spectrum; CHR-P = clinical high risk for psychosis. A = Reciprocal social interaction; B = Qualitative anomalies in communication; C = Restricted, repetitive and stereotyped behavior patterns; D = Developmental anomalies evident at/before 36 months.

**Table 9:** Analysis on the GAF scale, considering the scores relating to the current moment, the lowest in the past year, and the highest in the past year in the two groups.

	AS		CHR-P		Sign	r
	*M (SD)*	95%CI	*M (SD)*	95%CI	Mann-Whitney U Test	
GAF current	57.30 (15.8)	49.89–64.71	41.93 (15.9)	32.76–51.09	64.000 **	0.436
GAF low past year	55.95 (15.5)	48.67–63.23	37.43 (16.1)	28.12–46.74	49.000 ***	0.505
GAF high past year	63.20 (15.3)	56.03–70.37	51.57 (18.4)	40.93–62.21	88.500	0.324

** *p* ≤ 0.01; *** *p* ≤ 0.001. AS = autism spectrum; CHR-P = clinical high risk for psychosis. GAF = Global Assessment of Functioning.

**Table 10:** Analyses and comparisons of the three domains and the total of the ABAS-II in the two groups (considering the clinical cut-offs of the respective domains).

	AS		CHR-P		Sign.
	*n*	%	*n*	%	*χ²*
ABAS-II GAC (<cut-off)	10/20	50	3/13	23.07	2.392
ABAS-II DAC (<cut-off)	5/20	25	0/13	0	3.830 *
ABAS-II DAS (<cut-off)	17/20	85	5/13	38.46	7.679 **
ABAS-II DAP (<cut-off)	9/20	45	6/13	46.15	0.004

* *p* ≤ 0.05; ** *p* ≤ 0.01; AS = autism spectrum; CHR-P = clinical high risk for psychosis. ABAS-II = *Adaptive Behavior Assessment System*, Second Edition. GAC = total; DAC = conceptual domain; DAS = social dominance; DAP = practical domain.

**Table 11:** Analyses and comparisons of the three domains and the total of the ABAS-II in the two groups (without considering the clinical cut-offs of the respective domains).

	AS		CHR-P		Sign.	r
	M (SD)	95%CI	*M (SD)*	95%CI	Mann-Whitney U Test	
ABAS-II GAC	70.65 (13.1)	64.53–76.77	78.38 (9.9)	72.40–84.36	81.500	−0.315
ABAS-II DAC	79.65 (15.0)	72.62–86.68	87.92 (9.9)	81.94–93.91	88.000	−0.309
ABAS-II DAS	61.95 (9.5)	57.50–66.40	77.62 (14.0)	69.15–86.08	46.000 **	−0.547
ABAS-II DAP	76.05 (21.0)	66.21–85.89	73.00 (14.3)	64.34–81.66	126.000	0.084

** *p* ≤ 0.01. AS = autism spectrum; CHR-P = clinical high risk for psychosis. ABAS-II = *Adaptive Behavior Assessment System*, Second Edition. GAC = total; DAC = conceptual domain; DAS = social dominance; DAP = practical domain.

## Data Availability

The original contributions presented in the study are included in the article, further inquiries can be directed to the corresponding authors. The further data are not publicly available due to privacy.

## Abbreviations

Abbreviations	Definitions
CHR-P	Clinical High Risk for Psychosis
ADOS-2	Autism Diagnostic Observation Schedule, 2nd Edition
ADI-R	Autism Diagnostic Interview, Revised
SIPS/SOPS	Structured Interview for Psychosis Risk Syndromes/Scale for the Assessment of Prodromal Symptoms
SPI-CY	Schizophrenia Proneness Instrument—Child and Youth version
UHR	Ultra High Risk
APS	Attenuated Psychotic Symptoms
BLIPS	Brief Limited Intermittent Psychotic Symptoms
GRDS	Genetic Risk and Deterioration Syndrome
CAARMS	Comprehensive Assessment of at Risk Mental States
COPER/COGDIS	Cognitive/Perceptive Basic Symptoms/Cognitive Disturbances
DSM-5	Diagnostic and Statistical Manual of Mental Disorders, 5th edition
IQ	Intelligence Quotient
WISC-IV	Wechsler Intelligence Scale for Children, 4th edition
WAIS-IV	Wechsler Adult Intelligence Scale, 4th edition
K-SADS-PL	Kiddie-Schedule for Affective Disorders and Schizophrenia, Present and Lifetime Version
CSS	Calibrated Severity Score
SA	Social Affect
RRB	Restrictive and Repetitive Behaviors
GAF	Global Assessment of Functioning
ABAS-II	Adaptive Behavior Assessment System, 2nd edition
